# Adding Depth to Microplastics

**DOI:** 10.1021/acs.est.3c03620

**Published:** 2023-09-08

**Authors:** Margherita Barchiesi, Merel Kooi, Albert A. Koelmans

**Affiliations:** # Aquatic Ecology and Water Quality Management Group, Wageningen University, P.O. Box 47, 6700 DD, Wageningen, The Netherlands; $ DICEA—Department of Civil, Constructional and Environmental Engineering, Sapienza University of Rome, Via Eudossiana, 18, 00184 Roma, Italy

**Keywords:** Microplastics, Image analysis, Particle volume, Volume estimation models, Toxicologically relevant metrics

## Abstract

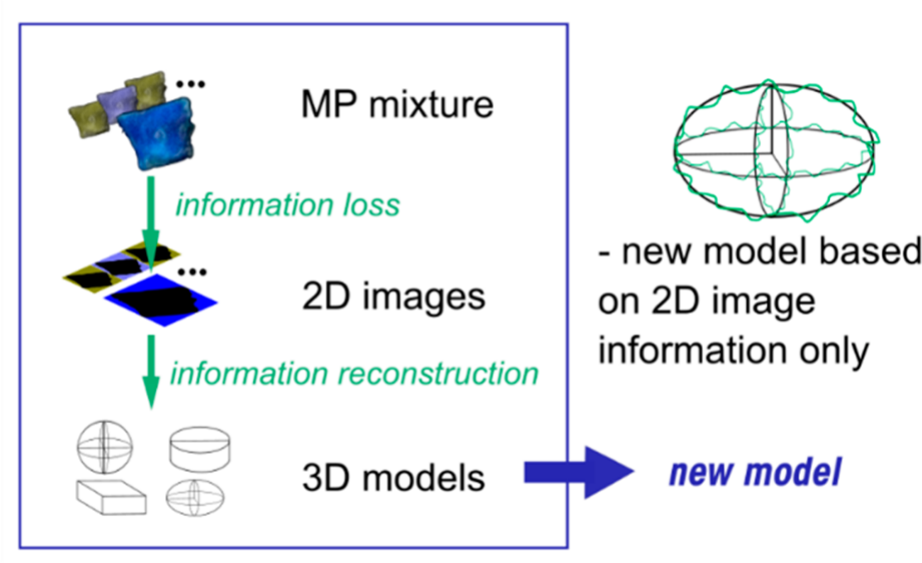

The effects and risks of microplastics correlate with three-dimensional (3D) properties, such as the volume and surface area of the biologically accessible fraction of the diverse particle mixtures as they occur in nature. However, these 3D parameters are difficult to estimate because measurement methods for spectroscopic and visible light image analysis yield data in only two dimensions (2D). The best-existing 2D to 3D conversion models require calibration for each new set of particles, which is labor-intensive. Here we introduce a new model that does not require calibration and compare its performance with existing models, including calibration-based ones. For the evaluation, we developed a new method in which the volumes of environmentally relevant microplastic mixtures are estimated in one go instead of on a cumbersome particle-by-particle basis. With this, the new Barchiesi model can be seen as the most universal. The new model can be implemented in software used for the analysis of infrared spectroscopy and visual light image analysis data and is expected to increase the accuracy of risk assessments based on particle volumes and surface areas as toxicologically relevant metrics.

## Abstract 2

Improved estimation of microplastic volumes from 2D image analysis data, aids microplastic risk assessment based on a food dilution effect mechanism

## Introduction

Our ability to accurately measure concentrations of microplastic particles (*MP*) and their characteristics is a prerequisite for monitoring spatiotemporal trends and risks of these particles to human health and the environment.^1−4^ To date, concentrations and characteristics of microplastic particles in our living environment are mainly determined using spectroscopic techniques.^5,6^ However, these techniques only provide information about the particles in two dimensions (2D), making it difficult to estimate properties that require all three dimensions (3D), such as particle volume, mass, and surface area.^7^ It is precisely these 3D properties that are necessary to properly determine the transport, effects, and risks of *MP*.^8−11^ For example, based on a growing body of evidence, recent risk assessments assume that the volume of particles ingested determines the risk posed by food dilution, whereas the risk of *MP* from translocation-mediated effect mechanisms is assumed to relate to the surface area of the particles.^4,12−14^ Here, the toxicological implications do not relate to the volume and area of individual particles, but to the collective volume and area of the bioavailable mixtures of particles as they occur in the air we breathe, in our food and drinking water, and in nature.^3,4,15,16^ Techniques that directly measure the properties of 3D particles, such as Raman imaging or AFM, are currently too labor intensive to be a viable option in the field of *MP* studies. Therefore, until better tools are available to start using those techniques more efficiently, it is crucial to accurately estimate the surface area and volume of environmental *MP* mixtures from 2D data. Only a very limited number of studies focus on estimating 3D characteristics, such as particle volume, based on 2D information and then verifying the findings with measurements of that 3D property.^17,18^ These studies mainly focus on evaluating the conversion from 2D to 3D information for individual particles. As mentioned, for risk assessment, the accuracy of estimating the volume or surface area of individual *MP* particles is of little importance, while knowing those characteristics for realistic mixtures of *MP* is more relevant, as only these realistic mixtures are found in the routes of exposure for humans and other biota. The accuracy of converting 2D data to 3D metrics for environmental *MP* mixtures has been provisionally evaluated by the work of Primpke et al. (2020),^19^ Tanoiri et al. (2021),^17^ and Isobe et al. (2019),^20^ but there are reasons to believe that the efficiency and accuracy of these estimates can be improved. For instance, relative errors will be smaller when larger particle numbers and masses are considered. Second, model inaccuracies that could be detectable on the individual particle level could cancel out when concerning higher particle numbers. Additionally, a wider range of measuring methods is available when assessing the bulk of a sample rather than a single small particle. Finally, the 2D–3D conversion methods published so far apply models containing empirical parameters calibrated on limited sets of data.^17,20,21^ This means that it cannot be assumed that the models have universal character. At the moment, they require recalibration for each new set of particles the models are applied to, which is labor-intensive. Two models do not require calibration,^18,22^ but these have hardly been verified.^18,19^ The question is whether modeling approaches are possible that do not require calibration and still come close to the accuracy that can be achieved with calibration-based models.

This work aims to further develop and validate mathematical models required for the conversion of 2D data to 3D parameters necessary for fate, effect, and risk assessment of environmentally relevant *MP*. The special aim of the latter is to avoid parameters that need calibration so that a potentially more universal model is obtained. Our second aim is to develop a new measurement method to estimate the collective volume of a mixture of environmentally relevant microplastic particles to evaluate existing and new models for the conversion of 2D to 3D data. We use volume displacement and pycnometer measurements to obtain volume data for various mixtures of individual *MP*, varying in shape, size, degree of weathering (surface roughness), and polymer type. Subsequently, existing models are evaluated and compared with the results of a new model that does not require calibration. The reliability of the models is thus evaluated on the “collective” volume of environmentally realistic *MP* in all its dimensions.

## Materials and Methods

### General Research Approach

Environmentally realistic *MP* (500–5000 μm) from Singapore and Netherlands beaches were used in this study. The mixture was divided into three categories (“Primary *MP*”, “Secondary *MP*”, and “Fibers”) based on *MP* shape. The categories “Primary *MP*” and “Secondary *MP*” were divided into two size groups: 1–2 mm and 2–5 mm by sieving. We assume that, for particles with equal spatial orientation, the conversion of 2D to 3D data after scaling on the dimensions of the particles is independent of those dimensions, just as the algorithm for conversion from radius to the volume of a sphere is the same regardless of the size of the sphere. Model calibration can then take place with easily measurable particles, but smaller and larger particles are also in the application domain. Three subsamples (batches) were taken for each group of each category. Three more subsamples with a lower number of *MP* per group (number of *MP* < 30) were also randomly selected for the categories “Primary *MP*” and “Secondary *MP*” to evaluate the relevance of the number of *MP* on the collective volume estimate. It was not possible to apply the same reasoning to fibers, due to difficulties in volume measurement. All these subsamples were then photographed and image analyzed to retrieve the 2D information. Their collective volume was then measured either with a pycnometer or by using a new method (see section Measuring Volumes of Diverse Microplastic Mixtures) for mixtures of particles too large to be analyzed by a pycnometer. Existing models from the literature were reviewed and evaluated concerning their ability to describe the data. A new model for improved estimation of volume is proposed. The model performances were statistically compared by applying the F-test.

### Overview of Approaches to Estimate Volume from Two-Dimensional Information

The strategy followed so far to estimate the volume of *MP* particles from 2D data is based on mathematical models. Here, we briefly discuss the main models used in the literature (Table 1).

**Table 1:**
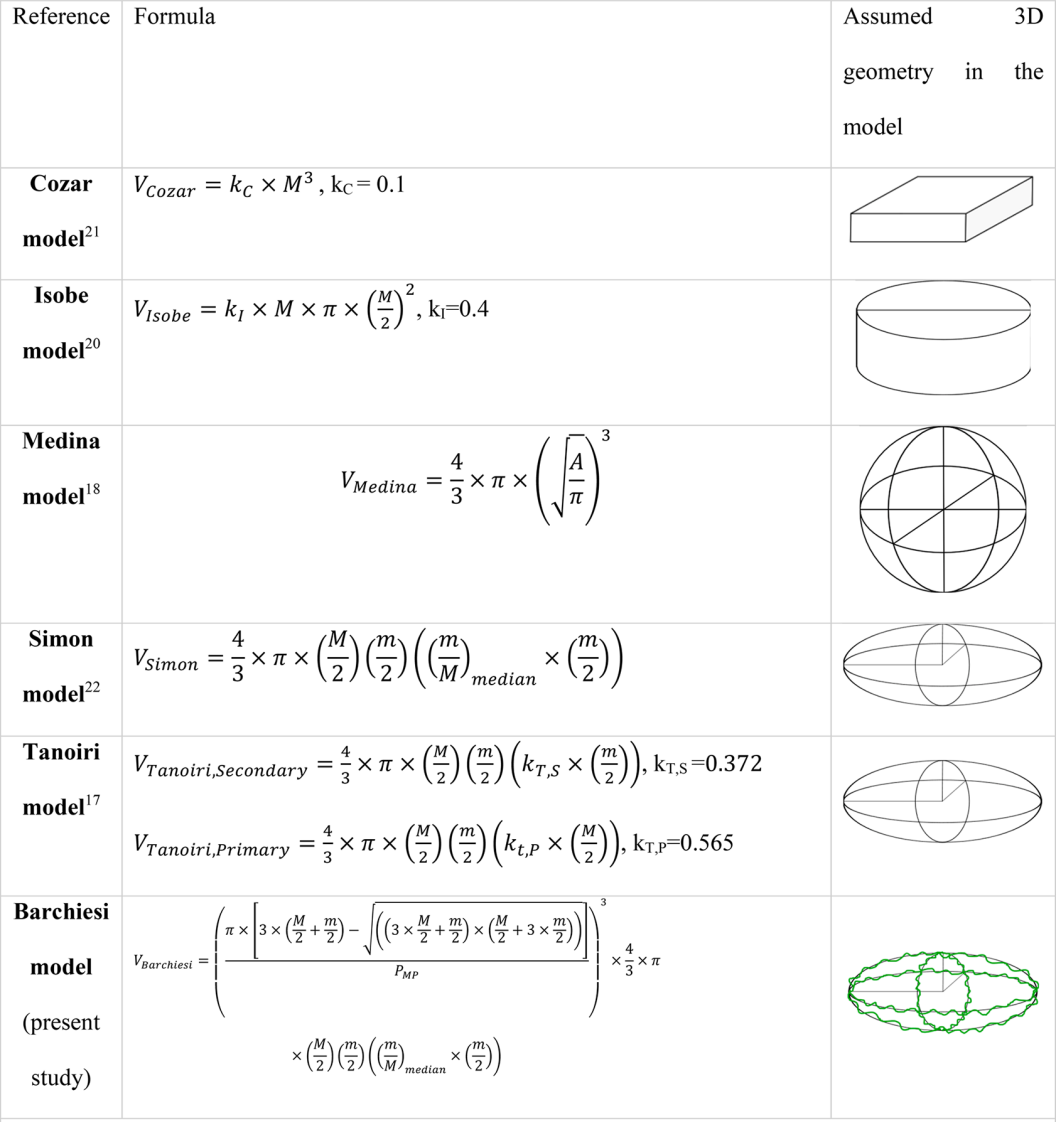
Overview of Models to Estimate Particle Volume from Two-Dimensional Metrics, Such As Area or Size^a^

a *V* = volume, *A* = area retrieved from 2D image analysis, *M* = major axis of the best-fit ellipse, *m* = minor axis of the best-fit ellipse, *P*_*MP*_ = perimeter of the **MP** from the 2D image, *k*_*i*_ = model calibration parameter. A more detailed explanation of how to use these models is provided as Supporting Information

#### Cozar Model

The geometric model by Cózar et al. (2014)^21^ assumes that the particles are cubic with equal sides with length *L* and a particle depth, or height: *H* = *K*_c_ × *L* The value for *K*_c_ was set to 0.1.^21^ The model has been developed to estimate the volume of ocean plastic *MP* and to compare it with measured data from ocean plastics, measuring 0.3–100 mm.^21^

#### Isobe Model

Isobe et al. (2019)^20^ modeled the particles as cylinders with a diameter equal to the particle length. Height (*H*) is assumed to be proportional to the length (*L*) by an adjustable shape factor between 0 and 1. This factor was estimated as *K*_I_ = 0.4 from calibration against field data obtained during their study. The study aimed to estimate the abundance of nonconservative microplastics in the upper ocean in the size range of 0.2–5 mm.

#### Medina Model

Medina Faull et al. (2021)^18^ proposed a model that assumes *MP* to be spherical with a diameter equal to that of a circle that has the same area as that of the particle in 2D (equivalent sphere diameter, ESD). Modeled volumes were compared with data obtained by Raman Imaging, for 31 particles, in a size range of 1 to 200 μm. The results were accurate with an *R*^2^ of measured vs estimated volume of 99%.

#### Simon Model

The most used model is that of Simon et al. (2018).^22^ Three assumptions are at the core of the model. First, the particles are assumed to have an ellipsoidal shape, with the major and minor axes on the XY plane calculated from the ellipse that best fits the particle projection on the XY plane (equivalent ellipse from the 2D area obtained by image analysis). Second, the particles are assumed to lie at their lowest energy state (therefore the *Z*-direction axis is the smallest dimension). Third, the asymmetry of the ellipsoid in the ZY plane is assumed proportional to that in the XY plane hence the axis in the Z direction (i.e., particle height “*H*”) is assumed to be in the same ratio to the 2D minor axis (“*W*” for width), as the 2D minor axis is to the 2D major axis (“*L*” for length) (i.e., *H*/*W* = *W*/*L*). The model was developed for mass balance in a wastewater treatment plant, for particles ranging from 10–500 μm. No validation was run.

#### Tanoiri Model

Tanoiri et al.(2021)^17^ applied the Simon model for large *MP* in the range of 1 to 5 mm, with a twist. They used two groups of *MP*, one for calibration and one for validation. The *z*-axis (particle depth or height) was estimated as a function of the major or minor axis of the best-fit ellipse. The function was derived with different empirical parameters for different *MP* groups. The *MP* groups were defined based on their shape and chemical characteristics. Due to the particle-specific calibration, good results were obtained with a total estimated mass deviating at most 3% from the actual mass of the *MP* sample used for validation. The model was applied to *MP* samples from a tidal flat at the mouth of the Tsurumi River (Japan). The calibrated parameters for calculating the *z*-axis of the ellipsoid for the *MP* groups referred to as “fragments” and “PE/PP pellets” by Tanoiri et al. (2021)^17^ were also used in our present study. The work of Tanoiri et al. (2021)^17^ also presents the first attempt at model comparison. However, they assess model reliability for mass, for *MP* in the 1–5 mm range, first measuring the mass of each particle individually.

#### Barchiesi Model

Here we present a new model based on the Simon model with an additional correction factor for surface irregularity. The correction factor is based only on the 2D information and does not require calibration with measured volume data (more details are in the following section).

The model trialed for fibers is that of Simon et al. (2018) which assumes a cylindrical shape and a 40% void fraction. The one proposed by Tanoiri et al. (2021)^17^ was not implemented due to the poor performance already shown by Tanoiri et al. (2021).^17^ The model proposed by Mintenig et al. (2020)^23^ that assumes a fixed fiber width of 15 μm was also not implemented being out of the size range studied.

### Volume Estimation from 2D Features without the Need for Calibration

All of the models proposed and described in the previous section theoretically require calibration, except for the Medina and Simon model. All of the parameters of the Medina and Simon model can be retrieved from the 2D images, while the other models require a “best fit” evaluation with measured volume data. However, this is not always possible or practical. Further conceptual model improvements can be made based on already-known model imperfections. For instance, it has been reported that the Simon model consistently overestimates *MP* volume or mass, especially for larger particles.^17,19^ The Simon model accounts for particle asymmetry regarding the major dimensions, that is, inequality of length, width, and/or height. However, there is a surface irregularity that remains unaccounted for. For instance, there is a residual lack of fit because the best-fit ellipse does not capture the actual irregularity in the 2D perimeter. If this irregularity exists in all three dimensions, then it will also affect the correctness of the estimated third dimension: particle depth. These irregularities at the surface relate to possible microvalleys, cracks, or -pores that are known to exist but are not detectable through 2D imaging.^24−26^ In 2D we only see a cross-section through micropores or valleys in the surface, whereas such cracks are not detected in 2D as long as they do not reach the perimeter.

Here we assume that the extent to which those irregularities exist in the 2D plane is quantified by the ratio between the best-fit ellipse perimeter, computed by the first Ramanujan approximation,^27^ and the actual perimeter of the *MP* observed in the image, and as provided by ImageJ.^28^ Realizing that these 2D irregularities are reflections of micropores and cracks that exist in 3D, this unitless correction factor should be applied to the three space dimensions and therefore is calculated to the power three (Barchiesi model, Table 1). The correction factor becomes less relevant as the *MP* is more regular. When the particle 2D area is a perfect ellipse or circle, the correction factor is equal to 1. This refined model is termed the Barchiesi model (Table 1), and the volume estimate *V*_*Barchiesi*_ can be summarized as *V*_*Barchiesi*_ = *C*_*f*_ × *V*_*Simon*_. The model equation is shown in equation 1, where “*M*” and “*m*” are the best-fit ellipse major and minor axes, respectively, and *P*_*MP*_ is the best-fit ellipse perimeter of the *MP* particle. Here, the first term refers to the correction factor (*C_f_*) that accounts for surface irregularity, whereas the other terms represent the volume of a perfectly regular ellipsoid as proposed by Simon et al. (2019)^22^ (Table 1).
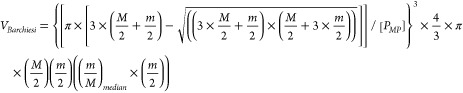
1

### Diverse Environmentally Realistic Microplastic Samples Used for Model Evaluation

Beached marine microplastic particles were collected in Singapore (Changi Beach) and The Netherlands (Hoek van Holland Beach). The plastic was sorted visually in the laboratory of Wageningen University by using forceps, and organic matter was removed. Separation of polymer versus nonpolymer particles was done visually, assisted by ATR-FTIR Spectroscopy (Agilent Cary 630). Additionally, expanded polystyrene (EPS) was removed via flotation in ethanol. Due to the origin of the particles, they represented an environmentally relevant and diverse mixture, especially in terms of particle size and shape. *MP* were fractionated by size using squared mesh sieves of 1 mm, 2 mm, and 5 mm. These fractions were subsequently divided into primary and secondary *MP* based on their shape: circular and pellet-shaped *MP* were categorized as “Primary *MP*”, whereas irregularly shaped *MP* were categorized as “Secondary *MP*”. Fibers were separated into a third group, which was not further fractionated concerning size. Primary *MP* were found only in the size fraction 2–5 mm. Three subsamples (batches) were taken from each size group of Primary and Secondary *MP* and from the fiber group to perform image analysis in triplicate (next section). The mass of *MP* for each batch varied in the range: 5.7–7.0 g for primary *MP*, 4–4.4 g for secondary *MP* in the size range 2–5 mm, 0.72–0.84 g for secondary *MP* in the 1–2 mm range, and 0.015–0.018 g for fibers. The number of particles varied between 200 and 325 particles.

Additionally, three to four *MP* were randomly selected for the size fraction between 2 and 5 mm or 16 to 30 for the size fraction between 1 and 2 mm. These are termed the “small samples”. These “small samples” were taken to evaluate the relevance of the number of *MP* on the collective volume estimate. All analyses were carried out in triplicate. Moreover, to analyze reproducibility, three “throws” were run for each batch. A “throw” is one deposition of *MP* on black carton, followed by Image analysis (see section “*MP* 2D characteristics”). The deposition of the particles was obtained by carefully shaking a small jar containing the particles close to the black carton until all of the particles were out and the jar emptied.

Theoretically, throws can lead to different results if particles are positioned differently while using the same mixture of particles. However, if the particles are always in their lowest energy state, with always the same *Z*-axis as the shortest dimension, minimal differences can be expected. Fibers were only thrown once due to their easy embrittlement.

**Figure 1:**
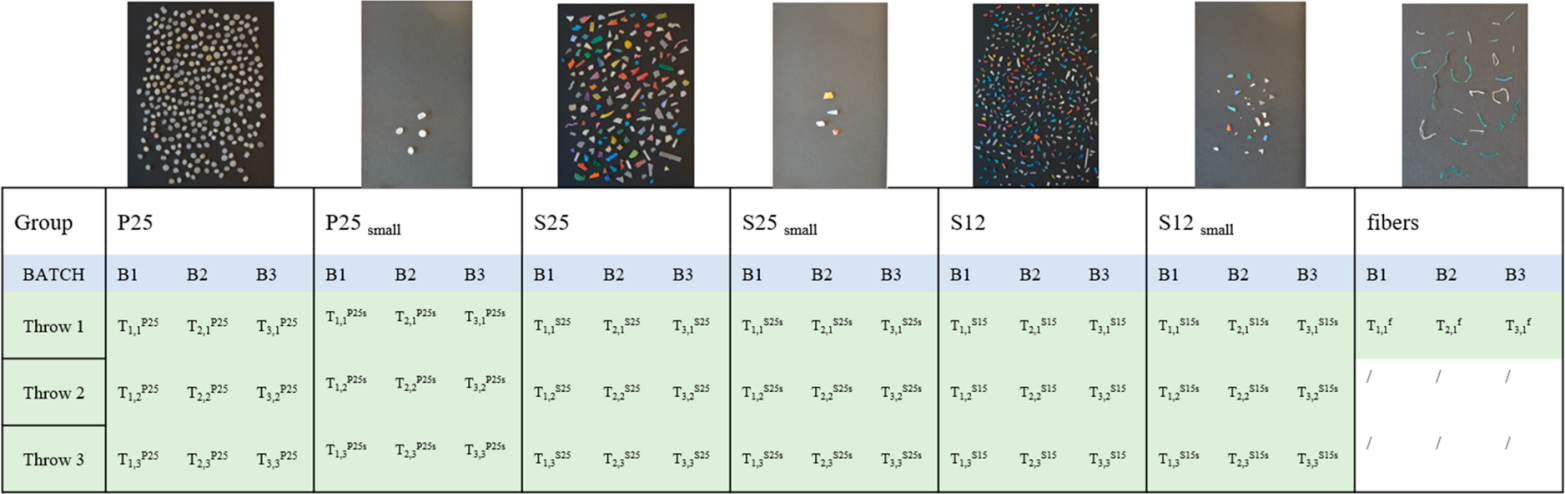
Subsampling and data organization (pictures not in scale). Code explanation: ‘P25’ primary *MP* in the size range of 2–5 mm, ‘S25’ secondary *MP* in the size range of 2–5 mm, and ‘S12’ secondary *MP* in the size range of 1–2 mm. Small samples are referred to as P25_small_, S25_small_, S12_small_ according to size. Only one throw was performed for fibers batches.

From here onward, we refer to primary *MP* in the size range of 2–5 mm as ‘P25’, secondary *MP* in the size range of 2–5 mm as ‘S25’, and secondary *MP* in the size range of 1–2 mm as ‘S12’. Small samples are referred to as P25_small_, S25_small_, S12_small_ according to size. The triplicates (batches) are from now on indicated by the index “*i*”, and throws (T) by the index “*j*”: e.g., the results from the “*j*^th^” throw of the “*i*^th^” replicate (batch) for group P25 is indicated as *T*_*i,j*_^P25^, as shown in Figure 1.

### Microplastic 2D Characteristics

The 2D data were acquired from the image analysis of high-resolution pictures of the *MP*. The pictures were taken with a 24.2 megapixel high resolution camera (Nikon D3200), positioned on a sturdy tripod, with a 35 mm or 105 mm focal length Nikon lens (depending on *MP* size), at ISO 200, using exposure times and aperture selected to accommodate the different light conditions during the day. Lens distortions, if any, were automatically corrected in-camera. The *MP* was scattered on a black carton. Picture postprocessing was done with the program NX Studio, openly available by Nikon.

The pictures were then analyzed with ImageJ.^28^ We developed a new macro that integrates the image thresholding plug-in with the image analysis plug-in to retrieve the 2D parameters area, perimeter, and length. Image quality was monitored by including a ruler and a reference object as an internal standard in each image. The image analysis process was modified for the analysis of fibers, to include the use of the plug-in “ridge detection”.^29,30^

2D *MP* characteristics were derived from the particle dimensions given by the ImageJ plug-in “analyze particles” (Figure 1).^28^ The dimensional parameters acquired are “Area”, ”Perimeter”, “Major axis”, “Minor axis”, and “Feret diameter”. “Major axis” and “Minor axis” represent the major and minor axis of the best-fit ellipse, which correspond to the parameters used by Tanoiri et al. (2021)^17^ (Figure S1 and S2). The Feret diameter is the longest distance between two points along the particle perimeter. Other parameters taken into consideration for *MP* characterization and comparison are the nondimensional “Circularity” and “Aspect Ratio”. Circularity corresponds to 4π × area/perimeter^2^ and Aspect Ratio is the ratio between the Major and the Minor axis of the best-fit ellipse (Figure S2). For all of the other parameters included in the models (*k*_*i*_, Table 1), the best-fit parameters obtained by the different studies presented in the section Overview of Approaches to Estimate Volume from Two-Dimensional Information were used. For particle “length”, the Major axis of the best-fit ellipse was used, following Tanoiri et al. (2021).^17^

The length and width of the fibers were obtained by the “ridge detector” plug-in in ImageJ.^30^ The Spearman correlation coefficient was used to evaluate the relation between the Feret Diameter and Circularity or Aspect Ratio.

### Measuring Volumes of Diverse Microplastic Mixtures

The volume of the *MP* mixtures was measured by two different methods, depending on the size and number of particles. A pycnometer (Ultrapyc 1500e by Quantachrome) was used to measure the collective volume of diverse particle mixtures in the size range of 1–2 mm, fibers, and small samples. The batches of diverse *MP* in the size range of 2–5 mm were not analyzable by the pycnometer since they were too big. Therefore a new method was developed. For these large particle samples, the collective volume was assessed by weight differences as follows. The weight (*W*_1_) of a volumetric flask filled with exactly 25.0 mL (*V*_1_) of ethanol was measured. Subsequently, in the same but empty flask, **MP** with known weight (*W*_*MP*s_) were added. Then, ethanol was added until a total (*MP* plus ethanol) volume of 25.0 mL was reached (*V*_2_). A narrow flask was used such that *V*_1_ can be assumed to be equal to *V*_2_, within acceptable error limits. The weight of the flask with *MP* and ethanol is measured (*W*_2_). The volume of *MP* was calculated from (detailed explanation provided as Supporting Information, Figure S3):
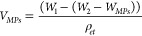
2in which, ρ_*et*_ is the density of ethanol. Ethanol was selected as the liquid of choice due to its lower density than most *MP* (ρ_*et*_ = 0.81 g/cm^3^, ethanol 96% acquired from VWR) which prevented the *MP* from floating at the surface. Furthermore, ethanol has a lower surface tension compared to water, which facilitates the release of air bubbles that may be present in the cracks of the *MP* and between the *MP*s and minimizes the occurrence of air pockets in and on the particles.

To maximize reproducibility, a narrow volumetric flask was used. The weights were measured with an analytical balance of ±0.0001 g precision at constant room temperature. The weight of the volumetric flask filled with ethanol and *MP* was taken after sonication for 15 min, to get rid of air bubbles possibly embedded at or within the *MP*. The volumetric flask was then left at rest for 30 min to return to room temperature (same temperature as the previous *W*_2_ weight measurements).

### Quality Assurance for the New Protocol for Volume Measurement and Image Analysis Automation

The reliability of the new volume measurement protocol was checked by testing the replicability of the volume measurement to validate the underlying hypothesis that *V*_1_ = *V*_2_. The weight of a 25.0 mL volumetric flask filled with ethanol was measured ten times, each time emptied and refilled. Moreover, the collective volume of glass spheres of 2 mm diameter was also measured both with the pycnometer and with the new protocol developed for particle volume measurement. These verification measurements were run in triplicates. The standard deviation of the 10 replicates of the weight measurements for the replicability of the volume acquisition was 0.016%, which is considered very good for the scope of this study. The difference between the glass spheres’ volume measurements run with the pycnometer and with the new ethanol volume displacement method, was less than 2%. The stability of the setup for picture acquisition was verified by including a reference object in each picture, as mentioned. The results of the automated image analysis were considered acceptable if the min–max difference of the measurement of the Area of the reference object was less than 1%. In all other cases, each image was processed singularly. The resolution was 18.5–19.2 pixels/mm for the 35 mm focal length and 42–44 pixels/mm for the 105 mm focal length camera lens. All the data were processed using R Studio or Microsoft Excel.

## Results and Discussion

### General Characteristics of the Studied Beached Plastic Particles

The sizes and shapes of the studied particles were highly diverse, with major axis lengths ranging from 30 μm to 22 mm, aspect ratios ranging from 1 to 13, and circularities ranging from 0.05 to 1 (Figure S4). Circularity decreased, whereas aspect ratio increased with increasing Feret diameter (Figure S4B, S4C), demonstrating that the smaller particles were more rounded, which was found earlier for marine plastic particles.^31^ Because full and accurate determination of polymer identity is not necessary for our goal of estimating particle volumes from 2D image data, this was limited. The main two polymers detected by ATR-FTIR on a subsample of 54 particles were PE and PP (33% and 67%, respectively).

### Performance of Models to Estimate *MP* Volume from 2D Image Analysis Data

The volumes measured by the pycnometer and by the newly developed protocol are compared to the modeled volumes based on measured particle dimensions in 2D (Table 1). The comparison considers the performance per group, per batch, and variability among throws. The performance of the models is evaluated using F-tests.

#### Barchiesi Model versus the Best Calibration-Based Model

We compared the performance of the calibration-based models with that of the calibration-free Barchiesi model. As mentioned, the calibration parameters are used as reported in the studies that proposed the models. Overall, the best performance is recorded for the Barchiesi model (present study), followed by the Tanoiri model and then the Simon model. The performance of all the models tested is provided in Table 2.

**Table 2:**
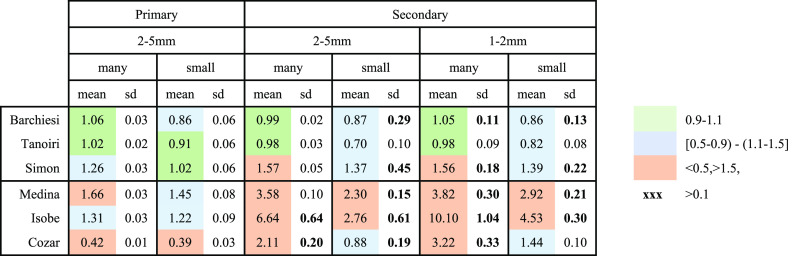
Results of Models Performance for *MP*s Volume Estimate as Average and Standard Deviation per Group of *V*_modeled_/*V*_measured_

Details on the two best-performing models, the Barchiesi and Tanoiri models, are reported in Table 3. The results confirm the ellipsoid as the best generic shape category for *MP* for the size range studied, albeit with the additional surface heterogeneity correction available only in the Barchiesi model. We observe that the Simon model overestimates particle volume up to 57%, which is in agreement with earlier findings^17,19^ (Table 2).

**Table 3:**
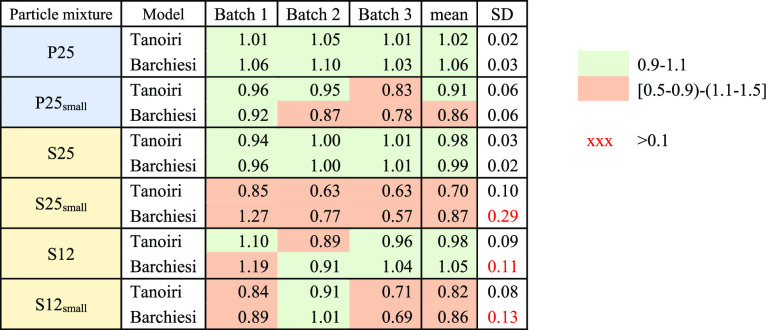
Quality of the Tanoiri and the Barchiesi Models in Estimating Particle Volume, Expressed As the Ratio of Modeled and Measured Volume, Shown Per Batch (Average of Throws), with the Mean and Standard Deviation among Batches^a^

a A ratio of 1 represents a perfect fit of the model to the measured data. “P” Primary *MP*s, “S” secondary *MP*s, “small” refers to samples of few particles (no. 3–16), “12”–“25” are the size range [1–2] mm and [2–5] mm respectively, as determined by sieving.

The calibration-free Barchiesi model performed well for the groups P25, S25, and S12, showing a group means *V*_modeled_/*V*_measured_ ratio in the interval [0.99–1.06] (Table 3). The performance of the Barchiesi model was also good on group mean for the samples with a lower amount of particles (*MP* n° 3–4 for P25_small_, S25_small_, and 26–33 for S12_small_), with a *V*_modeled_/*V*_measured_ ratio in the interval 0.86 to 0.87. The results per batch show *V*_modeled_/*V*_measured_ always in the interval 0.9–1.1 for the samples with many *MP*, except S12_B1 which records an overestimation of 20%. In the case of the small samples, the *V*_modeled_/*V*_measured_ per batch ranges from 0.57 to 1.27 (Table 3).

The Tanoiri model performed well for groups P25, S25, and S12, showing a group means *V*_modeled_/*V*_measured_ ratio in the interval 0.98–1.02 (Table 3). However, the accuracy dropped for secondary *MP* samples with few *MP*, showing a *V*_modeled_/*V*_measured_ ratio in the interval from 0.70 to 0.91. The results per batch show *V*_modeled_/*V*_measured_ always in the interval 0.9–1.1 for the samples with many *MP.* Regarding the small samples, the *V*_modeled_/*V*_measured_ per batch ranges from 0.63 to 0.96 (Table 3).

The standard deviation for the *V*_modeled_/*V*_measured_ ratio among batches per group is usually higher for small samples (Table 3).

Among the two best-performing models, Tanoiri and Barchiesi, the latter shows a slightly higher variability (Table 3). This means that the error per single batch might be somewhat higher, although on average it performs very well. The higher variability might be because there are no fixed (calibrated) parameters.

For the standard deviation among throws, the Barchiesi model shows a higher range compared to the Tanoiri model, with the smallest relative standard deviations of 0.38% up to 10.35%, while this range is 0.47%–2.55% for the Tanoiri model (Table S1). On this basis, there is therefore no clear preferred model. For the Barchiesi model, the highest variability values are recorded for the small samples, which can be explained in various ways. For instance, the Tanoiri model may be less sensitive to the particle orientation, which is a disadvantage if such differences exist. Another relevant aspect is the performance of the image thresholding algorithm, which might characterize the dimensions of *MP* differently if darker spots are present on different sides of the particle. However, the high particle number samples have a multiple throw standard deviation that is always less than 3% for both models (Table S1; Figure S5). Therefore, estimating the volume on a larger number of particles is more reliable, also taking into account the different possible orientations of the *MP* particles on the 2D plane and the performance of the image analysis algorithm.

The performance of the models decreased slightly with decreasing size for secondary particles, as we observed a higher standard deviation among batches for both the Tanoiri and the Barchiesi model. However, the performance on average is still in the *V*_modeled_/*V*_measured_ range of 0.9–1.1 which confirms that the 2D–3D conversion is independent of particle size, for the size range studied.

The relative performance of the two best-performing models was further assessed statistically by testing the statistical significance of the difference in residual errors of the modeled volumes (*V*_modeled_). Because the number of repetitions was low (three throws), the data was assumed to be normally distributed rather than that this could be formally demonstrated. Note that this does not necessarily disqualify the evaluation, as this can be compensated for by using a stricter *p*-level (see below).

The Barchiesi model did show a statistically significant better performance only for one batch in group S12_small_, whereas the Tanoiri model shows a statistically significant better performance only for one batch in group P25 (α < 0.05) (Table S3). To reduce the chance of Type II error due to the lack of demonstrated normality of data, the test was also run with α < 0.1. In this case, the results of the F test show a significantly better performance of the Barchiesi model, again only for one batch of group S12_small_ (α < 0.1) (Table S3). Instead, the Tanoiri model showed a significantly better performance in two batches of the group P25 and one batch P25_small_ (α < 0.1) (Table S3). However, in 88%, i.e., 31 of the 36 cases, neither model is preferable based on statistical criteria (Table S3). Therefore, it can be concluded that the Tanoiri model with the original calibrated parameters and the Barchiesi model without calibration parameters, have comparable performance in terms of the volume estimation of the particles analyzed in the present study.

The main difference between the two models is that the Tanoiri model contains a parameter that must be calibrated. Calibration is labor-intensive, especially if it is performed particle by particle. The good performances of the Tanoiri model for the particles used in the present study may also relate to the similar origin of the particles. The *MP* studied were, in both cases, beached *MP*, which might have been subdued to the same weathering processes and therefore show similar characteristics. This might not be the case for *MP* in other environmental compartments, for which specific calibration is due. However, the Barchiesi model only contains metrics derived from the 2D image analysis, has fewer, i.e., no calibration parameters, and, per Occam’s razor, can therefore be considered as the more universal and preferred model. Nevertheless, we recommend further testing of this claim.

#### Performance after Recalibration of Available Models

As mentioned, several of the evaluated models (Table 1) need to be calibrated to perform well. However, calibration is often a cumbersome and time-requiring step, especially if done on a particle-by-particle basis. We recalibrated the models based on the present collective volume data and compare the outcomes with the original parameter values (Table 4). The optimization was run on the results of each throw of each batch for every group. The Barchiesi model is not included because this model does not have calibration parameters.

**Table 4:**
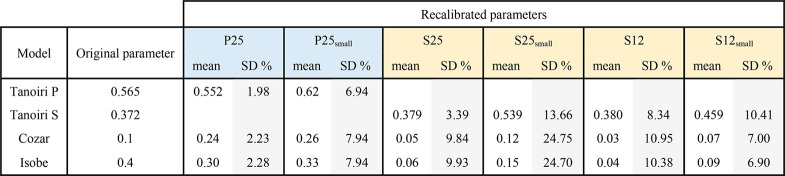
Results of the Parameter Optimization for the Tanoiri, Cozar, and Isobe Models^a^

a “Original parameters” relates to the reference value indicated by the authors in the original work.

It appears that for the Tanoiri model, there is a reasonably good agreement between the original and the reoptimized parameter values, for four of the six particle mixtures, with a maximum relative standard deviation of 14% (Table 4). However, for the Cozar and Isobe models, there are large differences between the original and the reoptimized parameter values, while the relative standard deviation is higher: up to 25%. These models thus require recalibration for each new data set, and their universal character is limited. Even within the same data set of particles, the optimal value for each throw varies significantly. This variation suggests the chance of significant error if the parameters are estimated for the whole data set from just one throw. Consequently, we recommend aiming for universal models as much as possible.

Regarding fibers, the model proposed by Simon et al. (2018)^22^ performed very well for two of the three samples, with a ratio of *V*_modeled_/*V*_measured_ of 1.07 and 1.1. However, the third sample showed an overestimation of about 45%. It should be noted that the measured volume of the fibers is much lower than the calibration volume for the pycnometer (0.01 vs 0.08 cm^3^). Therefore, the reliability of the measurement may not be optimal. Nevertheless, we present the results here for completeness, and they may be helpful for the future development of methods for fiber volume estimate validation.

### Implications and Outlook

We evaluated available models to convert 2D Image analysis data into estimates of the volume of realistic *MP* particle mixtures. Among the models evaluated, the best results in the size range analyzed are offered by those assuming an ellipsoidal shape as the best fit for the *MP* shape. The new Barchiesi model, which also accounts for irregularities in the ellipsoidal surface, showed a remarkably good fit for the particles studied. This is notable, as it does not require calibration, making it a relatively more universal model yet provides as good an approximation of measured particle volumes as the best available calibration-based model. It also removes the overestimation of the particle volume, which was often observed with the existing parameter-free model by Simon et al. (2018).^22^ Although we validated the model using a wide variety of environmentally relevant particles, we emphasize that the Barchiesi model is more universal than the other models only for the mixtures and types of particles studied here. The other models are also valuable, and it cannot be ruled out that they may work better in some other situations, for example, with individual particles. We also recommend further testing of the model, especially for smaller size classes. Our new and improved methods to measure and estimate the volume of microplastic mixtures based on 2D image analysis data can be incorporated into image analysis or IR spectroscopy data analysis software and can be used for the refinement of fate, effect, and risk assessments.

## Data Availability

All additional data is available on request.

## Supporting Information Available

Model performance results on *MP*s volume estimate as mean and standard deviation by group of *V*_modeled_/*V*_measured_; standard deviation in % of *V*_modeled_/*V*_measured_ among throws per batch; residual sum of square (RSS) errors of throws per batch by group; Feret length in relation to the major axis length averaged per group; *MP* 2D describing parameters; protocol for measuring volumes of diverse microplastic mixtures; the relationship between circularity, aspect ratio, and *MP* size; and data on reproducibility of the throws (PDF)
R code and ImageJ macros (TXT, TXT, TXT, TXT)
