# Supplementary material for: Adding Depth to Microplastics
Source: Environ Sci Technol. 2023 Sep 8;57(37):14015–23. doi: 10.1021/acs.est.3c03620 (PMC10515489; doi:10.1021/acs.est.3c03620)
Supplement: Supplementary file 1 — es3c03620_si_001.pdf [file es3c03620_si_001.pdf]

Supporting Information

Adding depth to Microplastics

Margherita Barchiesi[#,$], Merel Kooi[#], Albert A. Koelmans[#,*]

[#] Aquatic Ecology and Water Quality Management Group, Wageningen University, P.O. Box 47, 6700 DD, Wageningen, The Netherlands

[$] DICEA - Department of Civil, Constructional and Environmental Engineering, Sapienza University of Rome, Via Eudossiana, 18 – 00184 Roma

7 Pages

2 Tables

5 Figures

Results of models performance on MPs volume estimate

Table S1 Standard Deviation in % of $V_{modeled}/V_{measured}$ among throws per batch

	Model	batch1	batch2	batch3
P25	Tanoiri	0.63	0.72	0.81
	Barchiesi	0.76	0.38	0.52
P25small	Tanoiri	2.16	0.82	2.33
	Barchiesi	6.13	2.26	10.07
S25	Tanoiri	1.18	0.51	0.34
	Barchiesi	3.01	0.95	0.57
S25small	Tanoiri	1.00	1.12	2.55
	Barchiesi	2.94	5.94	1.07
S12	Tanoiri	2.21	1.69	0.75
	Barchiesi	0.59	2.19	2.05
S12small	Tanoiri	1.60	2.37	0.83
	Barchiesi	3.49	1.62	1.48

primary MPs
secondary MPs

>3%
<1%

Using the F-test to compare two models

We used the F-test to compare the models (Table S2). A formal limitation of this analysis is that it is difficult to confirm the normality of the residuals when the number of replicates is low (n=3). Therefore, the test was also run with a reduced significance ($\alpha < 0.1$) to reduce the chance of type II error and to balance out the uncertainty on the normality condition. In this case, the outcomes do not change relevantly compared to $\alpha < 0.05$, with the Tanoiri model performing better for two batches in the group P_25 and one batch S12_small. The Barchiesi model performs better for one batch in group S25_small and one batch in group S12_small. However, in the far majority of the cases, i.e. in 32 of the 36 cases, neither model is preferable based on statistical criteria.

Table S2 Residual sum of square (RSS) errors of throws per batch by group.

	**RSS**	**batch1**	**batch2**	**batch3**
**P25**	Tanoiri	2.92E-02**	4.60E-01	1.82E-02*
	Barchiesi	5.27E-01	1.63E+00	1.29E-01
**P25_small**	Tanoiri	7.75E-05	7.70E-05*	6.69E-04
	Barchiesi	3.26E-04	6.29E-04	1.24E-03
**S25**	Tanoiri	2.20E-01	3.00E-03	4.59E-03
	Barchiesi	1.58E-01	6.27E-03	1.33E-02
**S25_small**	Tanoiri	1.82E-04	1.84E-03	2.45E-03
	Barchiesi	6.49E-04	7.43E-04	3.24E-03
**S12**	Tanoiri	1.86E-02	2.05E-02	4.11E-03
	Barchiesi	5.65E-02	1.88E-02	3.28E-03
**S12_small**	Tanoiri	2.53E-04	1.59E-04	1.65E-03
	Barchiesi	1.19E-04	5.63E-06**	1.89E-03

*** statistically significant better performance for alpha <0.05, *statistically significant better performance for alpha <0.1.*

Additional supporting results on MP characteristics

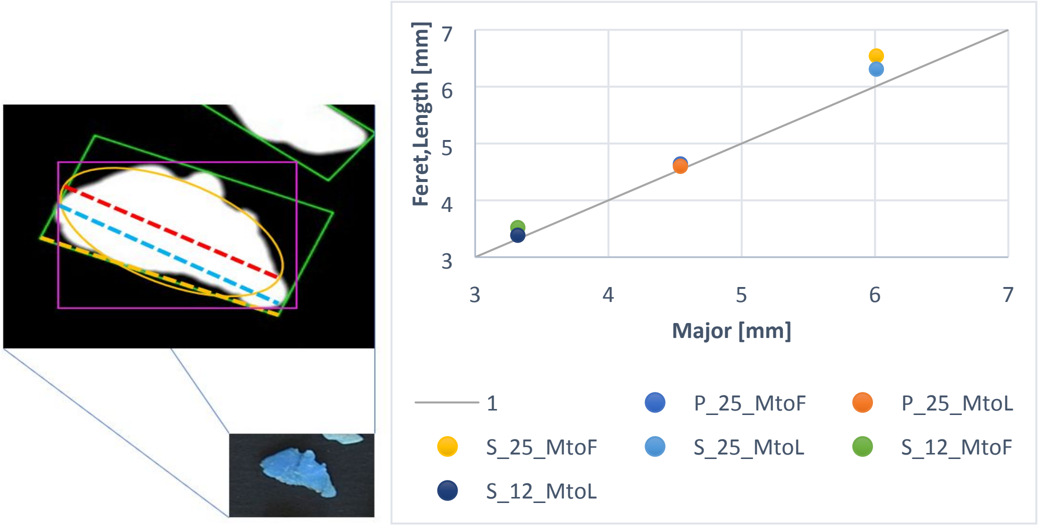

Figure S1. Feret length in relation to the major axis length averaged per group. MtoF "Major axis to Feret", MtoL "Major axis to Length". Whereas MP length is usually considered as a given, there are different proxies that are used to indicate the length of a particle:

1. Feret diameter or maximum caliper: the longest distance between two points on the perimeter of the particle (*blue dotted line*)
2. Major axis: major axis of the best-fit ellipse (*red dotted line*)
3. Length based on the tilted box: longest side of a rectangular box that contains the particle, with sides not parallel to x-y reference axis (*yellow dotted line*)
4. Length as the y parallel side of the bounding box (*pink line*); these are the length and width as indicated by ImageJ.

None of these descriptors fit with the diagonal of the square meshes usually used to separate particles lengthwise. The most used shape descriptors are reported in Figure S2, including an example from the MP used in this research, comparing the averages by MP group.

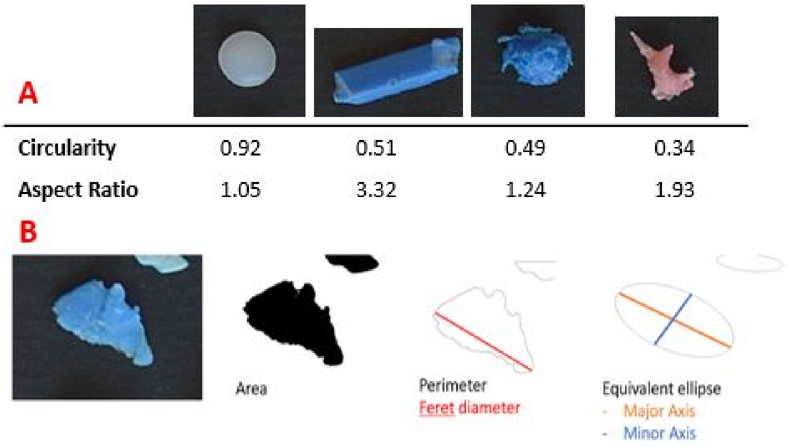

Figure S2. MP 2D describing parameters. (A) Shape descriptors circularity and aspect ratio. (B) size-related metrics area, perimeter, and equivalent ellipse.

Protocol for measuring volumes of diverse microplastic mixtures

The new protocol for volume measurements for MPs samples not analyzable with a pycnometer develops as follows (scheme showed in Figure S1):

- the weight of the MP sample is acquired (W_{MP});
- the weight of a volumetric flask filled with exactly 25.0ml (V_1) of ethanol is measured (W_1);
- subsequently, in the same but empty flask, MP with known weight (W_{MP}) are added;
- then, ethanol is added until a total (MP plus ethanol) volume of 25.0 ml is reached (V_2). A narrow flask is used such that V1 can be assumed to be equal to V2, within acceptable error limits;
- the weight of the flask with MP and ethanol is measured (W_2).

Hp: V_1=V_2

W_{MP}= weight of MP

W_1= weight of 25.0 ml flask with only ethanol

W_2= weight of 25.0 ml flask with ethanol and MPs

A = W_1-W_{MP}= weight of the volume of ethanol needed to fill the bottle at 25.0ml when plastics are present

W_2-A =weight of the ethanol with the same volume of the plastics

$V_{MPs} = \frac{(W_1 - (W_2 - W_{MPs}))}{\rho_{et}}$= volume of the plastics

in which, ρ_{et} is the density of ethanol. Ethanol was selected as the liquid of choice due to its lower density than most MPs (ρ_{et} = 0.81g/cmc, ethanol 96% acquired from VWR) which prevented the MP from floating at the surface. Furthermore, ethanol has a lower surface tension compared to water, which eases the release of the bubbles and minimizes the occurrence of air pockets in/on the particles.

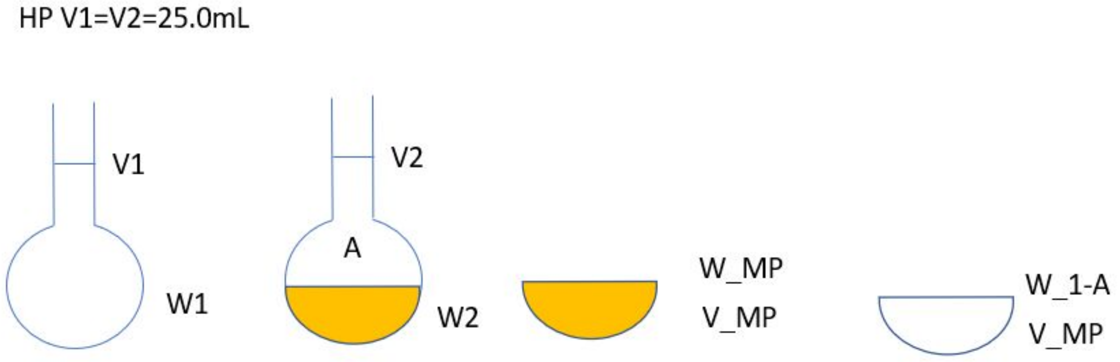

Figure S3 Schematic representation of MP volume measurement strategy, V= volume, W= weight

The relationship between Circularity, Aspect Ratio, and MP size

To investigate the relationship between Circularity, Aspect Ratio, and MP size (as represented by the Feret diameter), we created a dataset with the dimensions of all the MPs included in the study. We also noted whether each MP belonged to the "primary" or "secondary" group (reported as 'P' and 'S' in S4, respectively). We then grouped the MPs by size based on their Minor axis dimension, as shown in Figure S4B-C. Note that the size grouping is slightly different from that obtained by sieving, as some MPs with a Minor axis > 5mm were present due to the squared mesh of the sieves used to separate the particles (the size of the diagonal is 7mm). Primary MPs did not show any particular relation to size and occupied a small area of the x-y plane. However, secondary MPs exhibited a more regular and less elongated shape with decreasing size in each group, as circularity decreased with increasing Feret diameter (Fig. S4B), while Aspect ratio increased with increasing Feret diameter (Fig. S4C). These findings suggest a correlation between MP shape and size.

To test the hypothesis of a possible correlation between the parameters, we evaluated the Spearman correlation factor for Feret diameter and the parameters under investigation, namely Circularity and Aspect Ratio. For primary MPs, the correlation was either not significant (p-value > 0.005) or very weak (Spearmans' rho < 0.19). However, the correlation was significant for all secondary MPs groups (p-value < 0.005). The Feret-Circularity correlation factor for secondary MPs ranged between -0.6 and -0.8, whereas the range for the Feret-AR correlation was between 0.5 and 0.9. The correlation was also significant for the entire dataset (p-value < 0.05). It is important to keep in mind that the p-value of the correlation depends on several parameters, including the number of data points available. In this case, we had a large number of data points (around 1000). Nevertheless, the data support the hypotheses of a more regular and less elongated shape with decreasing size for secondary MP.

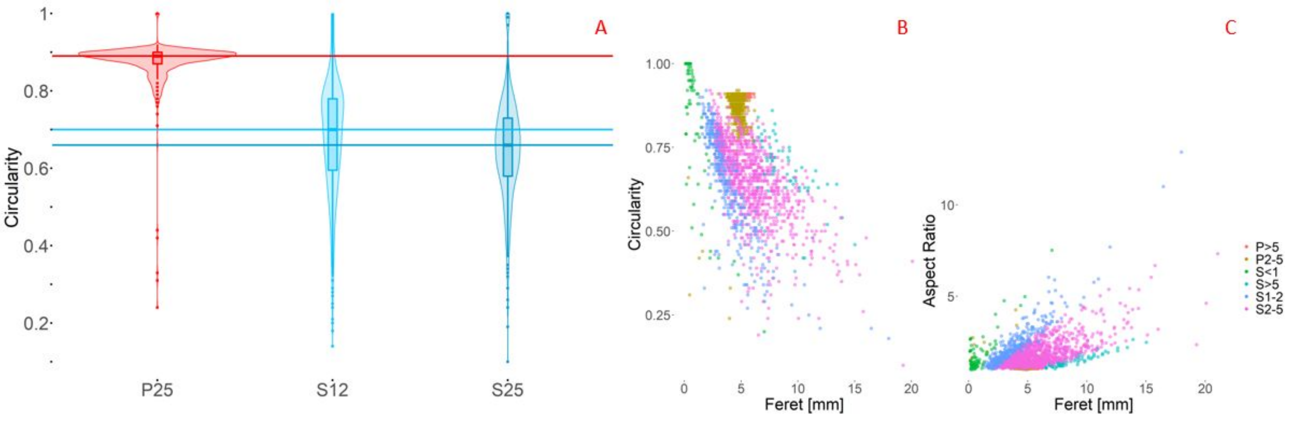

Figure S4 (A) Violin plot of parameter Circularity per MPs type and size group. Red and Blue lines are the median for each group. (B-C) Scatterplot with MPs grouped by colors by Minor Axis.

Testing the reproducibility of the throws

To characterize MP, empirical cumulative frequency distributions (ECDF) of the major axis were first drawn to understand the reproducibility of the throws: the ECDFs for each throw overlap almost perfectly for samples with many MP (Figure S4). This confirms the insignificance of particle orientation and location in the image. An increase in frequencies for MP in the lower size range for the 3rd throw of the sample with fewer MPs is observed (Figure S4B), which may be explained by the fragmentation of fragile particles that occurred during the three throws. However, this mechanism only plays a role when only a few particles are involved.

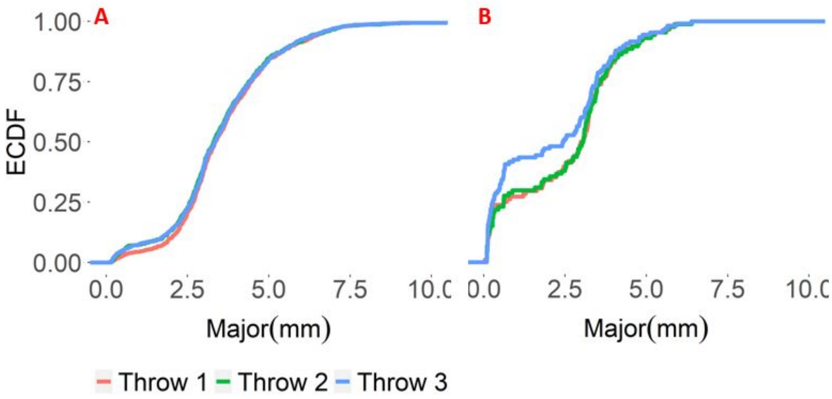

Figure S5 Example ECDF batches in 1-2mm size group: ECDF A average n° particles 300, ECDF B average n° particles 28. (small sample).'Major' is the major axis of the best fit ellipse.

Moreover, Primary and Secondary MP in the size range 2-5mm can be distinguished by the parameter "Circularity" with the threshold set at about 0.8 (outliers aside), as shown in Figure S4A. It seems that, for secondary MP, circularity increases with decreasing size, implying that these MP gain a more "rounded" shape when they get smaller. That smaller secondary plastic particles are more rounded was also recently reported from a latent class analysis of MP sampled from the ocean surface (Alkema et al., 2022).
